# Syndrome de Takotsubo dans sa forme Inversée Secondaire à une Envenimation Scorpionique Grave: À Propos D'un Cas

**DOI:** 10.48327/PWX0-M245

**Published:** 2021-01-26

**Authors:** A. Ben Jemaa, M. Bahloul, H. Kallel, O. Turki, M. Dlela, M. Bouaziz

**Affiliations:** 1 Département de soins intensifs, Hôpital universitaire Habib Bourguiba, 3029, Sfax, Tunisie; 2 Faculté de Médecine de Sfax. Université de Sfax, Boulevard Majida Boulila, Sfax 3029, Tunisie

**Keywords:** Syndrome de Takotsubo, envenimation scorpionique, Réanimation, hôpital, Sfax, Tunisie, Maghreb, Afrique du Nord, Takotsubo syndrome, scorpion envenomation, Intensive care unit, hospital, Sfax, Tunisia, Maghreb, Northern Africa

## Abstract

L'envenimation scorpionique (ES) est un motif d'admission fréquent en réanimation. La sévérité du tableau clinique est liée essentiellement à l'atteinte cardio-respiratoire. En effet, l'état de choc cardiogénique et l'oedème pulmonaire aigu sont les principales causes de décès après une ES. Cependant, la nature de la cardiomyopathie liée à l'ES fait toujours l'objet des débats. Le syndrome de Takotsubo au cours de l'ES, bien qu'il puisse aider à mieux élucider la physiopathogénie de cette cardiomyopathie a été exceptionnellement rapportée. Nous décrivons un cas de syndrome de Takotsubo dans sa forme inversée chez un patient âgé de 26 ans admis dans le service de réanimation médicale pour une envenimation scorpionique grave. Son évolution a été favorable. En conclusion, au cours de l'ES, l'atteinte cardiaque remplit tous les critères cliniques et paracliniques du syndrome de Takotsubo, soulignant l'importance de la décharge catécholaminergique. Nous discutons la prise en charge thérapeutique de ce syndrome dans cette situation particulière.

## Résumé

Scorpion envenomation (SE) is common in tropical and subtropical regions. Cardio-respiratory manifestations, mainly cardiogenic shock and pulmonary oedema are the leading causes of death after scorpion envenomation. Cardiac failure can be due to massive release of catecholamines, myocardial damage induced by the venom or myocardial ischemia. Although it has been exceptionally reported, Takotsubo syndrome during SE can help to better elucidate the pathophysiology of this cardiomyopathy. We report a case of inverted Takotsubo following a SE in a 26-year-old patient admitted to the Intensive care unit department for severe scorpion envenomation. His evolution was favorable. We concluded that cardiac involvement in this case fulfills all clinical and paraclinical criteria of Takotsubo syndrome emphasizing the importance of catecholaminergic discharge during scorpion envenomation. We discuss again the management of this syndrome in this specific condition.

## Introduction

L'envenimation scorpionique (ES) est un motif d'admission fréquent en réanimation dans les zones tropicales et subtropicales [8]. La sévérité du tableau clinique est liée essentiellement à l'atteinte cardio-respiratoire. En effet, l'état de choc cardiogénique et l'oedème pulmonaire aigu (OAP) sont les principales causes de décès après l'ES [6]. La nature de la cardiomyopathie liée à l'ES fait toujours l'objet de plusieurs débats. En effet, au moins trois mécanismes à savoir la myocardite adrénergique, la myocardite toxique et l'ischémie myocardique, ont été incriminés [5]. En effet, la décharge importante de catécholamines, joue un rôle fondamental dans la genèse de la dysfonction systolique du ventricule gauche (VG) au cours de l'ES [7]. Le syndrome de Takotsubo est caractérisé par un dysfonctionnement systolique transitoire se manifestant par une ballonisation de l'apex ou par des troubles de la cinétique des segments médians basaux ou focale du ventricule gauche [12, 13]. Ce syndrome exceptionnellement rapporté dans la littérature, peut aider à mieux comprendre la physiopathologie de l'atteinte cardiaque au cours de l'ES [1, 15]. Nous décrivons un cas de syndrome de Takotsubo inversé suite à une ES grave.

## Cas Clinique

Il s'agissait d'un patient âgé de 26 ans admis au service de réanimation médicale pour oedème aigu pulmonaire (OAP) et état de choc cardiogénique secondaire à une ES grave (stade 3) par un scorpion identifié comme *Androctonus australis*, rapporté mort par les parents.

**Fig. 1:**
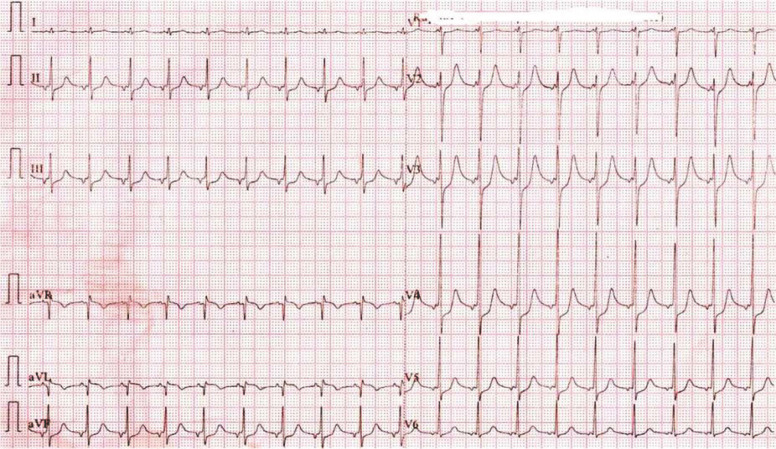
L'électrocardiogramme fait à l'admission montrant un sus décalage de ST en latéral haut avec un sous décalage en antérieur et en inférieur The electrocardiogram done on admission shows a higher ST shift in upper lateral with a lower ST shift in anterior and inferior

**Fig. 2:**
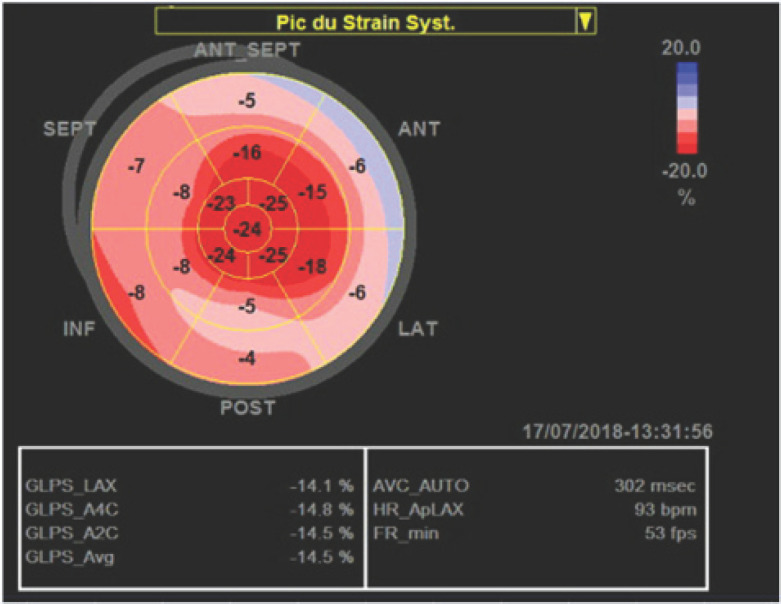
L2DStrain longitudinal du ventricule gauche, montrant une nette diminution de la contractilité longitudinale prédominante sur les territoires basaux et sa normalité dans les segments apicaux Longitudinal 2DStrain of the left ventricle, showing a clear decrease in the predominant longitudinal contractility in the basal territories and its normality in the apical segmentsand inferior

**Fig. 3:**
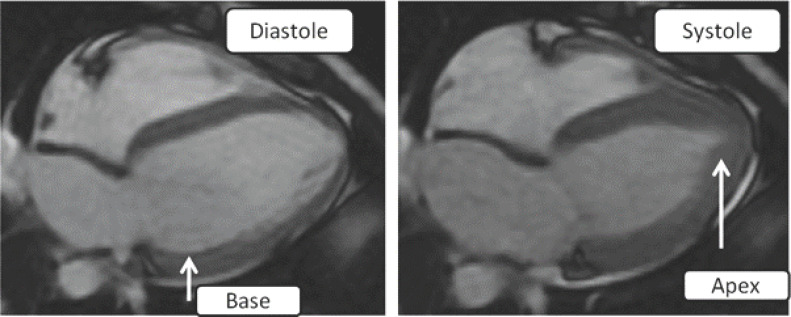
L2DStrain IRM cardiaque montrant une dysfonction systolique du ventricule gauche prédominante au niveau des segments basaux avec une bonne contractilité apicale Cardiac MRI showing predominant left ventricular systolic dysfunction in the basal segments with good apical contractility

L'examen initial trouvait une pression artérielle à 80/40 mm/Hg avec une fréquence cardiaque à 120 bpm et une froideur des extrémités. L'électrocardiogramme montrait un sus décalage de ST en latéral haut avec un sous décalage en antérieur et en inférieur (Fig. 1). Le dosage des troponines ultrasensibles était à 2,9 ng/ml, l'échocardiographie transthoracique (ETT) montrait une altération de la fonction systolique du VG (FE2D = 33%) avec une akinésie des segments basaux. Un complément par étude du strain longitudinal a montré une altération de la déformation myocardique des segments basaux (Fig. 2). L'IRM cardiaque montrait une dysfonction systolique du VG prédominante au niveau des segments basaux (Fig. 3) avec absence de rehaussement tardif au gadolinium, en faveur du syndrome de Takotsubo. Le patient a été mis sous Dobutamine à la dose de 10 μg/kg/min. Son évolution a été marquée par la stabilisation de l'état clinique avec un sevrage progressif des catécholamines. Il est sorti après 6 jours. Le contrôle échocardiographique après 15 jours a montré une récupération d'une fonction systolique VG normale.

## Discussion

Le syndrome de Takotsubo est une entité caractérisée par un dysfonctionnement systolique transitoire, se manifestant par une ballonisation de l'apex ou par des troubles de la cinétique des segments médians, basaux ou focaux du ventricule gauche [12, 13]. Ce cas clinique répond aux critères diagnostiques les plus récents pour le diagnostic du syndrome de Takotsubo [12, 13]: des anomalies régionales de la cinétique segmentaire du VG caractéristiques entrainant une dysfonction systolique VG réversible à l'échocardiographie, la notion du stress physique ou émotionnel (décharge adrénergique secondaire à l'ES), des anomalies à l'électrocardiogramme, une élévation modérée des biomarqueurs sériques, des dommages myocardiques et l'exclusion du diagnostic de la myocardite infectieuse par l'IRM cardiaque.

Le syndrome de Takotsubo partage des caractéristiques cliniques et paracliniques communes avec le syndrome coronarien aigu (SCA) avec ou sans sus-décalage du segment ST ainsi qu'une mortalité hospitalière comparable, ce qui peut être expliqué par l'altération de la microcirculation coronaire secondaire à l'hyperstimulation du système sympathique [11]. Le syndrome de Takotsubo est comparable à un SCA microvasculaire [12, 13]. Il peut partager en partie la même physiopathologie évoquée au cours de l'ES, avec l'orage adrénergique [5], mais sans l'ischémie myocardique [4] avec des constatations cliniques et paracliniques similaires. La description du syndrome de Takotsubo au cours de l'ES n'a été rapportée dans la littérature que par l'équipe de Miranda [15] et celle d'Abroug [1], alors que la liaison entre l'ES et l'orage adrénergique avait été établie depuis longtemps.

Notre observation concerne un cas illustrant un syndrome de Takotsubo dans sa forme atypique (inversée), c'est-à-dire présentant une dysfonction basale et latérale au lieu d'être prédominante à l'apex du ventricule gauche comme dans le syndrome typique. Cela a une implication clinique et pronostique: la forme inversée est décrite chez des patients plus jeunes souffrants de comorbidités neurologiques [12, 13]. La dysfonction systolique du VG est beaucoup moins sévère que dans la forme typique, ainsi que l'élévation du facteur natriurétique de type B. La forme inversée a un meilleur pronostic avec un taux de létalité plus bas a un an [12, 13].

Le traitement actuel de l'OAP cardiogénique et de l'état de choc au cours de l'ES est basé sur les inotropes [2], parmi lesquels les catécholamines et la dobutamine. Etant donné qu'au cours de l'ES on assiste à une réponse inappropriée aux catécholamines, ces derniers doivent être utilisés avec précaution et d'autres alternatives thérapeutiques doivent être préférées. En effet, une létalité de 20% a été rapportée chez les patients atteints du syndrome de Takotsubo traités par des catécholamines [16]. De plus, l'infusion de catécholamines a été incriminée dans l'induction et l'aggravation de la ballonisation apicale observée au cours du syndrome de Takotsubo [3, 14]. Récemment, il a été suggéré que le lévosimendan pourrait être utilisé efficacement et en toute sécurité dans le syndrome de Takotsubo en tant qu'inotrope alternatif aux catécholamines [18]. Devant la décharge massive catécholaminergique au cours du syndrome de Takotsubo, l'utilisation des bêtabloquants semble raisonnable jusqu'à la récupération complète de la fonction systolique du ventricule gauche, mais il n'existe pas d'études cliniques à l'appui de cette hypothèse. Des expériences sur des animaux ont montré que la ballonisation est atténuée après l'administration des médicaments bloquant les récepteurs alpha et bêta adrénergique [17, 19].

Les inhibiteurs de l'enzyme de conversion ou les antagonistes des récepteurs de l'angiotensine II peuvent potentiellement faciliter la récupération de la fonction systolique du VG [16]. Cependant, l'utilisation des bêtabloquants et des inhibiteurs de l'enzyme de conversion, peut s'avérer risquée initialement chez les patients qui se présentent avec un tableau d'envenimation scorpionique grave avec état de choc cardiogénique et chez qui sont généralement associés un état de choc cardiogénique et un oedème pulmonaire secondaires à une altération sévère de la fonction systolique [2, 5]. Dans cette situation, la dobutamine a prouvé son efficacité et améliore la plupart des perturbations hémodynamiques de l'envenimation grave, avec un effet bénéfique sur la mortalité [10]. La fréquence de l'accès hypertensif au cours de l'ES est très variable (allant de 10 à 77% selon les séries) [2, 5]. Cette hypertension est le plus souvent éphémère et survient précocement après la piqûre, pour laisser la place à un état de choc cardiogénique au bout de 30 à 60 min [2, 5].

La décharge catécholaminergique au cours du syndrome de Takotsubo peut être à l'origine d'une activation plaquettaire pouvant influencer la présentation clinique et déterminer le pronostic des malades atteints du syndrome de Takotsubo [9]. Une étude rétrospective récente a démontré que l'utilisation de l'aspirine ou de la double anti-agrégation plaquettaire était associée à un taux faible d'événements cardiovasculaires indésirables majeurs [9]. Cependant, l'ES grave se caractérise essentiellement par une vasoconstriction des artères coronaires [4] et, en dehors de la présence des facteurs de risque cardiovasculaire, nous pensons que cette prescription est inutile.

Finalement, Notre observation confirme que l'atteinte cardiaque dans notre cas remplit tous les critères cliniques et paracliniques du syndrome de Takotsubo, soulignant l'importance de la décharge catécholergique au cours de l'ES. La principale limite est l'absence de réalisation de coronarographie. Cependant, l'IRM cardiaque a permis d'éliminer une origine ischémique.

## Conclusion

L'atteinte cardiaque dans ce cas remplit tous les critères cliniques et paracliniques du syndrome de Takotsubo soulignant l'importance de la décharge catécholergique au cours de l'ES. Le défi est de mieux caractériser l'atteinte cardiaque au cours de l'ES afin d'améliorer la prise en charge et le pronostic des patients victimes de ce type d'envenimation.

## Conflits D'intérêts

Les auteurs ne déclarent aucun conflit d'intérêts.
